# Ultra-Processed Foods and Inflammatory Bowel Disease: A Narrative Review of Epidemiology, Mechanisms, and Dietary Implications

**DOI:** 10.3390/nu17243852

**Published:** 2025-12-10

**Authors:** So Yoon Choi, Won Moon

**Affiliations:** 1 Department of Pediatrics, Kosin University Gospel Hospital, Kosin University College of Medicine, Busan 49267, Republic of Korea; ks200546@kosinmed.or.kr; 2 Department of Internal Medicine, Kosin University Gospel Hospital, Kosin University College of Medicine, Busan 49267, Republic of Korea

**Keywords:** ultra-processed foods, inflammatory bowel disease, food additives, diet, nutrition therapy

## Abstract

Ultra-processed foods (UPFs), industrial formulations rich in refined substrates and additives, have been increasingly examined as plausible contributors to gut dysbiosis and mucosal inflammation relevant to inflammatory bowel disease (IBD). This narrative review synthesizes epidemiological, mechanistic, and interventional evidence on UPF intake and IBD based on a structured literature search from 2010 to 2025. Large-scale prospective cohorts consistently associate higher UPF intake with increased risk of Crohn’s disease (CD), whereas findings for ulcerative colitis (UC) remain weaker or inconsistent. Among individuals with established IBD, observational data suggest that greater UPF consumption correlates with higher disease activity and relapse, although potential confounding and reverse causation must be considered. Preclinical studies demonstrate that specific UPF constituents—including emulsifiers, carrageenan, maltodextrin, microparticles, and excess dietary salt—can disrupt epithelial barrier integrity, alter the gut microbiota, and activate immune pathways, providing biological plausibility while underscoring translational gaps. Interventional evidence, particularly for exclusive enteral nutrition and the Crohn’s Disease Exclusion Diet, suggests clinical benefit from reducing UPFs or selected additives, mainly in CD, though data in adults and UC remain limited. Overall, current evidence indicates that dietary strategies to limit UPF exposure may represent a promising and modifiable component of IBD management. Future research should prioritize standardized exposure assessment, mechanism-based human trials, and personalized nutrition approaches to refine clinical applicability.

## 1. Introduction

Inflammatory bowel disease (IBD), encompassing Crohn’s disease (CD) and ulcerative colitis (UC), represents a group of chronic, relapsing inflammatory disorders of the gastrointestinal tract whose global incidence continues to rise. Although genetic susceptibility contributes to disease risk, the rapid increase in IBD in newly industrialized countries underscores the critical role of environmental and lifestyle factors in disease pathogenesis [1]. Among these, diet has emerged as one of the most significant and modifiable determinants of disease onset and course.

Over the past few decades, the global dietary landscape has undergone a profound transition, marked by the pervasive consumption of ultra-processed foods (UPFs). According to the NOVA classification, UPFs are industrial formulations composed primarily of refined ingredients such as sugars, starches, hydrogenated oils, and protein isolates combined with cosmetic additives designed to enhance flavor, texture, and shelf life [2]. Common examples include sugar-sweetened beverages, packaged snacks, processed meats, and ready-to-eat meals. UPFs currently account for more than half of total caloric intake in many high-income nations and are rapidly expanding across low- and middle-income regions [2,3,4].

Epidemiological studies increasingly implicate high UPF intake as a risk factor for several chronic conditions, including obesity, metabolic syndrome, type 2 diabetes, cardiovascular disease, and certain cancers [5]. More recently, large-scale prospective cohorts, such as the Prospective Urban Rural Epidemiology (PURE) study, the U.S. and the UK Biobank, have examined UPF consumption in relation to IBD risk [1,6,7]. While findings vary across populations, a consistent trend has emerged linking high UPF intake with an increased risk of CD, whereas associations with UC appear weaker or absent [1,6,7]. While findings vary across populations, many studies report a positive association between higher UPF intake and increased CD risk, whereas associations with UC appear weaker or inconsistent. These observations require cautious interpretation, given potential confounding by socioeconomic status, lifestyle factors, or reverse causation (e.g., individuals with prodromal symptoms modifying their diet before diagnosis).

Beyond disease onset, UPFs may also impact disease activity and relapse rates among patients with established IBD [8,9]. Mechanistic investigations using animal models provide biological plausibility for these observations: additives such as emulsifiers (e.g., carboxymethylcellulose, polysorbate-80) and carrageenan have been shown to disrupt the intestinal barrier, alter the gut microbiota, and promote mucosal immune activation [10,11]. However, translation of these findings to humans is limited by differences in dosing, exposure duration, and species-specific physiology.

Given the growing global burden of IBD and the feasibility of dietary modification as a preventive and therapeutic approach, elucidating the relationship between UPFs and IBD is an urgent research priority.

This narrative review synthesizes current epidemiological evidence linking UPF intake with IBD risk and activity, examines potential mechanistic pathways while acknowledging translational limitations, and discusses the clinical implications of dietary strategies that reduce UPF exposure.

**Figure 1:**
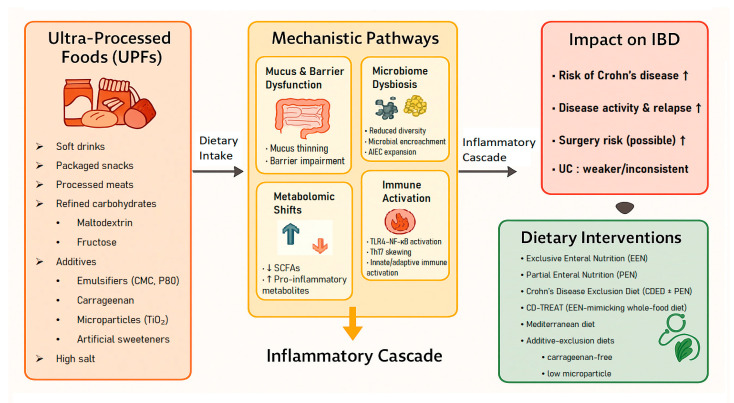
Conceptual framework linking ultra-processed foods (UPFs) to mechanistic pathways, inflammatory cascade, clinical outcomes, and dietary interventions in inflammatory bowel disease (IBD).

A conceptual overview of the proposed links between UPF components, mechanistic pathways, inflammatory responses, and clinical outcomes is presented in Figure 1.

## 2. Methods

### 2.1. Study Design

This article is a narrative review designed to synthesize epidemiological, mechanistic, and interventional evidence regarding the impact of UPFs on IBD. While this review was not registered in PROSPERO as it is not a systematic review, we followed a structured and transparent approach appropriate for narrative synthesis, ensuring a comprehensive and reproducible selection of the literature.

### 2.2. Search Strategy

We conducted a comprehensive literature search using the electronic databases PubMed/MEDLINE, Embase, and Scopus for articles published from 1 January 2010, to 31 March 2025. To systematically identify evidence corresponding to the review’s three main thematic areas (epidemiology, mechanisms, and therapeutics), we employed a multi-step search strategy:

- Primary Search: We broadly searched for combinations of “ultra-processed foods,” “NOVA classification,” and “inflammatory bowel disease” (including “Crohn’s disease” and “ulcerative colitis”).
- Targeted Secondary Search: Based on initial findings, we performed targeted searches for specific UPF-associated components identified in the literature, such as “food additives,” “emulsifiers” (e.g., carboxymethylcellulose), “carrageenan,” and “maltodextrin.”
- Reference Screening: We manually screened the reference lists of retrieved articles and relevant systematic reviews to identify additional pivotal studies not captured by the initial electronic search.

We did not apply automated filters for study design or outcomes during the electronic search in order to avoid inadvertently excluding mechanistically important or emerging interventional studies. Instead, relevance was assessed during manual screening as outlined in Section 2.3.

### 2.3. Eligibility Criteria

Studies were selected based on inclusion criteria: human studies (cohort, case–control, RCTs) and relevant mechanistic models (in vivo/in vitro) defining UPF intake or additive exposure. Studies were required to provide clearly defined exposure measures (e.g., NOVA classification or additive doses relevant to dietary intake). Exclusion criteria included studies focusing solely on single nutrients without the context of processing, or irrelevant animal models.

### 2.4. Study Selection and Synthesis

Data were selected through a qualitative review process. Titles and abstracts were initially screened for relevance to the core themes (epidemiology, mechanisms, therapeutics). Full texts of potentially relevant articles were then assessed based on the inclusion criteria. Consistent with narrative review methodology, study selection emphasized conceptual relevance and biological plausibility rather than quantitative pooling. Priority was given to recent high-quality cohorts, mechanistic studies demonstrating biological plausibility (e.g., specific additive effects), and interventional trials, while redundant or non-specific studies were excluded.

Finally, evidence was synthesized qualitatively and organized into three thematic domains: (1) epidemiological associations, (2) mechanistic pathways, and (3) clinical and therapeutic implications.

## 3. Epidemiology

The relationship between UPF consumption and IBD has been examined in multiple large-scale prospective cohorts and patient-based studies. Collectively, evidence points to a consistent association between higher UPF intake and increased risk of CD, whereas the relationship with UC remains weaker or inconsistent [6,7,12,13]. Despite differences in study design, exposure definitions, and population characteristics, the signal for CD appears robust. However, interpreting these findings requires careful consideration of methodological variations, including potential misclassification of dietary exposures and residual confounding.

Among individuals with established IBD, observational studies suggest that higher UPF intake is linked to greater disease activity and an increased risk of relapse, extending the association beyond disease onset [8,9]. Additional studies that examined Westernized or processed dietary patterns, although not explicitly using the NOVA classification, provide complementary evidence. Diets rich in fats, sweets, processed meats, and sugar-sweetened beverages are associated with an increased risk of IBD, particularly CD, whereas higher dietary fiber intake (especially from fruits) appears protective [14,15,16,17].

### 3.1. Prospective Cohorts

The major prospective cohort studies evaluating UPF intake and incident IBD are summarized in Table 1. The PURE study followed 116,087 adults across 21 countries for nearly 10 years. Individuals consuming ≥5 servings/day of UPFs had a significantly higher risk of IBD than those consuming <1 serving/day (HR 1.82, 95% CI 1.22–2.72), with the strongest associations for soft drinks, processed meats, and salty snacks [1].

In three large U.S. cohorts (NHS I, NHS II, and HPFS; >200,000 participants; up to 20 years of follow-up), higher UPF intake was significantly associated with CD risk in a dose—response manner, but not with UC [6]. In contrast, the NutriNet-Santé cohort (France; 105,832 adults; mean follow-up 2.3 years) found no significant association between UPF proportion and incident IBD. This null finding may reflect limited statistical power due to short follow-up duration and a small number of incident cases [12].

In the UK Biobank, participants in the highest tertile of UPF intake had approximately a twofold higher risk of CD (HR 2.00, 95% CI 1.32–3.03) but no association for UC. Notably, higher UPF consumption also predicted a fourfold increased risk of IBD-related surgery, suggesting a possible role in disease progression [7].

### 3.2. Patient Cohorts

Patient-based studies evaluating UPF intake among individuals with established IBD are summarized in Table 2. In Israel, a cross-sectional study of 242 patients with established IBD found that higher UPF intake was associated with active disease (OR 3.82, 95% CI 1.49–9.8), while minimally processed foods were inversely associated [8]. In a subsequent prospective cohort of 111 CD patients in remission, higher UPF intake predicted nearly a fourfold increased risk of relapse at one year (HR 3.86, 95% CI 1.30–11.47) [9]. These findings extend the epidemiologic signal beyond disease onset to disease activity and relapse. However, these observational findings warrant caution due to the possibility of reverse causation, where individuals with prodromal symptoms or active flares may preferentially consume softer, processed foods to minimize gastrointestinal discomfort, thereby complicating interpretation of temporality.

### 3.3. Meta-Analyses

Several systematic reviews and meta-analyses have consolidated the epidemiologic evidence linking UPF intake with IBD. Narula et al. performed the first quantitative synthesis combining seven observational studies and reported a consistent positive association between UPF consumption and CD (pooled RR 1.70, 95% CI 1.24–2.35), with less consistent findings for UC [13]. Babaei et al. conducted a large-scale dose–response meta-analysis including more than four million participants, confirming that higher UPF intake was significantly associated with increased overall IBD risk (pooled RR 1.28, 95% CI 1.11–1.48) [19].

Together, these quantitative syntheses strengthen the evidence that UPFs represent a modifiable dietary risk factor for IBD, particularly for CD. In addition, a complementary umbrella review has broadly linked dietary and nutritional exposures, including processed food consumption, to the incidence and progression of IBD, thereby providing a broader context for these quantitative findings [18]. This broader evidence base underscores how UPF-rich dietary patterns, embedded within global nutritional and cultural transitions, may contribute to the rising incidence of IBD worldwide.

### 3.4. Complementary Evidence from Dietary Pattern Studies

Several studies that did not explicitly use the NOVA classification nevertheless support a link between processed dietary patterns and IBD. In a Canadian pediatric case–control study, imbalances in fatty acid intake and low consumption of fruits and vegetables were associated with increased CD risk [14]. A follow-up analysis in the same cohort showed that dietary patterns rich in fast foods and sugar-sweetened beverages were linked to higher pediatric CD risk [15]. Likewise, a multicenter Case–Control study in Japan (*n* = 579 cases, 1246 controls) reported that higher intake of sweets, fats, and oils was associated with increased IBD risk, consistent with a Westernized dietary pattern [16].

In addition, systematic and prospective evidence indicates that diets high in total fat, *n*-6 polyunsaturated fatty acids, processed meats, and refined sugars are associated with greater IBD risk, whereas higher dietary fiber, particularly from fruits, appears protective against CD [17,20].

### 3.5. Synthesis and Methodological Considerations

Taken together, evidence from multinational cohorts, meta-analyses, and patient-based studies consistently indicates that higher consumption of UPFs increases the risk of developing CD and may exacerbate disease activity or relapse in established IBD.

However, significant uncertainties regarding causality and disease specificity remain. The stronger associations observed for CD compared with UC may reflect plausible biological differences, including the greater exposure of the small intestine where CD predominantly manifests to dietary emulsifiers and additives, whereas the colon’s thicker, double-layered mucus barrier may offer relative protection [6,7,21].

Furthermore, potential confounding factors must be acknowledged. Socioeconomic status (SES) strongly influences UPF consumption, with lower SES often linked to higher intake; simultaneously, higher SES has historically been associated with increased IBD risk, complicating the direction of association [1,22]. Genetic susceptibility also plays a role, as host genetics influence microbiome composition and responses. In addition, dietary exposure misclassification remains a concern, as food frequency questionnaires may inadequately capture additive-rich foods or processing levels. Finally, reverse causation remains a critical concern, particularly in patient-based studies, as individuals with undiagnosed or active IBD may self-restrict fiber-rich whole foods in favor of processed alternatives to manage symptoms [6,8].

Overall, while the epidemiologic signal is robust for CD, future research must employ designs that better account for these confounders and utilize standardized exposure measures to clarify the causal role of UPFs.

## 4. Mechanistic Pathways

UPFs are distinguished by their composition of refined substrates such as added sugars and starches and a wide array of industrial additives including emulsifiers, thickeners, colorants, and non-nutritive sweeteners. These components collectively alter host–microbe interactions and disrupt intestinal homeostasis. Emerging experimental and translational evidence delineates at least four interrelated mechanisms through which UPFs may promote intestinal inflammation: (i) erosion of the mucus layer and impairment of epithelial barrier integrity; (ii) gut microbial dysbiosis characterized by bacterial “encroachment” and expansion of pathobionts; (iii) pro-inflammatory metabolomic alterations, including reductions in short-chain fatty acids (SCFAs); and (iv) activation of innate and adaptive immune pathways, particularly involving TLR–NF-κB and Th17 signaling axes.

Together, these processes create a biologically plausible, though not yet definitively causal, framework linking UPF consumption to the onset and progression of IBD. A conceptual schematic summarizing the major biological pathways through which UPFs may influence intestinal inflammation is presented in Figure 1.

### 4.1. Emulsifiers (Carboxymethylcellulose, Polysorbate-80)

In murine models, chronic exposure to the dietary emulsifiers carboxymethylcellulose (CMC) and polysorbate-80 (P80) leads to thinning of the protective mucus layer, microbial encroachment toward the epithelium, reduced microbial diversity, and the development of colitis in genetically susceptible hosts; even wild-type mice display low-grade inflammation and metabolic disturbances under prolonged exposure [10]. Mechanistically, these alterations are accompanied by closer bacteria–epithelium proximity and an increased pro-inflammatory potential of the gut microbiota [10].

Translational relevance has been demonstrated in humans: in a randomized controlled feeding trial in healthy adults, supplementation of CMC (15 g/day) to an additive-free diet significantly altered the gut microbiota composition, reduced fecal SCFAs, and increased postprandial abdominal discomfort, providing human proof-of-principle for emulsifier-induced dysbiosis and barrier dysfunction [23]. However, this trial was small and short-term, limiting generalizability to broader clinical populations.

### 4.2. Carrageenan (CGN)

CGN exposure activates the TLR4–Bcl10–NF-κB signaling axis and induces IL-8 secretion in human intestinal epithelial cells [24,25,26]. Comprehensive reviews suggest that CGN may trigger multifaceted innate and adaptive immune responses, and animal studies demonstrate CGN-associated dysbiosis, including reduced Akkermansia muciniphila [27,28]. Disruption of epithelial tight-junction proteins has been reported even at food-grade concentrations [29]. At concentrations relevant to typical dietary exposure, CGN also causes cell-cycle arrest and epithelial stress responses [30]. In murine models, carrageenan administration (e.g., 10–50 mg/kg/day via drinking water) induces colitis, with significantly reduced severity observed in Bcl10-deficient mice, underscoring the role of the Bcl10–NF-κB pathway in mediating inflammation [31]. Translationally, a double-blind randomized trial in patients with ulcerative colitis in remission demonstrated that carrageenan supplementation (200 mg/day) accelerated clinical relapse compared with placebo, suggesting that CGN restriction may be beneficial in susceptible individuals [32]. While clinically meaningful, this study was limited by small sample size, and larger trials are required to confirm the effect.

### 4.3. Refined Carbohydrate Additives: Maltodextrin and Added Sugars

Maltodextrin (MDX), widely used in UPFs, enhances biofilm formation and adhesion of adherent-invasive *E. coli* (AIEC), a CD-associated pathobiont [33], and impairs antimicrobial defenses in intestinal epithelial cells and macrophages [34]. In IL-10^−/−^ mice, chronic MDX exposure increases colitis severity, reduces goblet cells, and promotes microbial encroachment into the mucosa, suggesting impaired mucus-mediated protection [35,36]. Additional work indicates that MDX may also interfere with autophagy pathways that are relevant to CD susceptibility genes, although these findings remain primarily experimental and require human validation.

Beyond MDX, diets enriched in added sugars—particularly fructose—have been shown to worsen colitis in murine models by disrupting epithelial tight-junction integrity and altering gut microbial composition [37,38]. These effects appear modifiable, as switching to a standard chow diet or adding psyllium restores SCFA-linked pathways and improves inflammatory outcomes. However, the translational relevance of these findings is limited by the high fructose doses commonly used in animal studies and by differences in human dietary patterns and food-matrix contexts.

### 4.4. Non-Nutritive Sweeteners (NNS)

NNS, widely used in UPFs, have been shown to alter the gut microbiota and modulate metabolic responses in humans. In a recent controlled trial, several commonly consumed NNS produced person-specific impairments in glycemic control, driven by individual microbiome configurations and accompanied by microbiota-dependent functional alterations [39]. Earlier foundational work demonstrated that NNS could induce glucose intolerance in mice and in subsets of healthy human volunteers, with the effect fully transferable via microbiota transplantation, confirming a causal, microbiome-mediated mechanism [40]. Although these studies do not assess IBD directly, they illustrate how UPF-associated additives can reshape host–microbe interactions in ways potentially relevant to mucosal inflammation.

### 4.5. Microparticles and Colorants (Titanium Dioxide, E171)

Food-grade titanium dioxide (E171) contains micro- and nanoparticles that can interact with the intestinal barrier and alter gut microbial composition in experimental models [41,42,43]. These particles have been shown to accumulate within intestinal mucus, modulate microbial biofilms, and influence epithelial and immune responses [44].

Regulatory assessments reflect ongoing uncertainty regarding the safety of E171. In 2021, the European Food Safety Authority (EFSA) concluded that titanium dioxide could no longer be considered safe as a food additive due to unresolved concerns related to potential genotoxicity, leading to its removal from the European Union food market in 2022 [45,46]. In contrast, the U.S. Food and Drug Administration (FDA) continues to permit E171 as a food colorant. This regulatory divergence is noteworthy, particularly because several U.S. food products—including candies, confectioneries, and brightly colored processed snacks marketed to children—contain higher relative amounts of titanium dioxide, resulting in proportionally greater exposure per body weight in younger populations.

However, despite these safety concerns, direct clinical evidence linking titanium dioxide to the onset or progression of IBD remains limited. Existing findings are derived largely from in vitro studies and animal models, and the doses and exposure forms used may not fully reflect real-world dietary conditions. As such, while E171 is biologically plausible as a contributor to mucosal dysfunction, its specific role in human IBD pathogenesis has not been definitively established and warrants further investigation.

### 4.6. High Salt as a UPF-Linked Property

Dietary salt, which is typically elevated in many UPF formulations, has been implicated as an independent modifier of intestinal inflammation. In murine models, high-salt diets exacerbate chemically induced colitis, characterized by reductions in Lactobacillus species, depletion of SCFA, and impaired barrier-related gene expression [47,48]. Additional studies demonstrate that excessive salt intake promotes Th17 differentiation and worsens 2,4,6-trinitrobenzenesulfonic acid (TNBS)-induced colitis through immune-mediated pathways [49,50]. Although these findings do not establish a direct causal link between dietary salt and IBD onset in humans, they suggest that salt-rich dietary patterns, common in UPFs, may potentiate mucosal inflammation in susceptible hosts.

### 4.7. Translational Gaps and Limitations

Although experimental models provide compelling evidence for the pro-inflammatory effects of individual UPF components, several important limitations constrain direct translation to human IBD. Many animal studies involve continuous, high-dose additive exposures (e.g., emulsifiers or carrageenan in drinking water) that may not mimic the intermittent, mixed-matrix consumption patterns typical of human diets. Species-specific differences such as thinner mucus layers, faster intestinal transit, and distinct microbial communities in mice further influence host responses to dietary additives.

Moreover, human evidence remains limited. Most randomized controlled trials evaluating UPF additives include small sample sizes (e.g., 16 participants in the CMC feeding study) and short exposure durations, and they rarely assess clinical IBD endpoints. Observational associations in humans may also be influenced by confounding factors, including socioeconomic status, genetic susceptibility, comorbid dietary patterns, and potential reverse causation, in which early symptoms prompt patients to shift toward softer, more processed foods.

Thus, while the mechanistic data provide biological plausibility, establishing a causal relationship between specific UPF components and human IBD will require long-term, adequately powered clinical studies using standardized exposure measurements.

### 4.8. Integrated Mechanistic Synthesis

Across experimental models and, increasingly, human intervention studies, UPF-characteristic ingredients and processing properties show a reproducible ability to disrupt intestinal homeostasis. These effects include: (i) erosion of the mucus layer and impairment of epithelial barrier integrity, (ii) induction of dysbiosis and microbial encroachment, including the expansion of pathobionts such as AIEC, (iii) reductions in SCFAs and broader metabolomic disturbances, and (iv) activation of innate and adaptive inflammatory pathways, including TLR4–NF-κB and Th17-mediated responses. Collectively, these mechanistic pathways provide a coherent biological framework supporting the epidemiologic signal linking UPF consumption and IBD risk, while also emphasizing the need to interpret current evidence within its translational limitations. Representative mechanistic evidence supporting these pathways is summarized in Table 3.

## 5. Therapeutic Implications

Growing epidemiological and mechanistic evidence has renewed interest in dietary interventions that reduce UPFs intake or eliminate specific UPF-associated additives as potential therapeutic strategies for IBD. While nutrition counseling is increasingly recognized as an integral component of comprehensive IBD care, the quality and robustness of evidence supporting each dietary approach differ substantially. At present, the strongest and most reproducible clinical evidence exists for CD, particularly in children, whereas data for UC remain limited, heterogeneous, and often exploratory. As a result, dietary strategies must be interpreted within the context of available clinical trials, mechanistic plausibility, and patient-centered feasibility.

### 5.1. Exclusive Enteral Nutrition (EEN) and Partial Enteral Nutrition (PEN)

EEN, which replaces the habitual diet entirely with a nutritionally complete formula for 6~8 weeks, remains the recommended first-line induction therapy for pediatric CD according to ECCO/ESPGHAN guidelines [51]. Beyond its established clinical efficacy, EEN is hypothesized to exert benefit partly through the removal of habitual dietary exposures, including UPFs and common food additives, while providing controlled, antigen-light nutrition [52,53]. However, real-world implementation remains challenging due to palatability issues, psychosocial burden, and declining adherence over time.

PEN, which provides a proportion of calories from formula alongside whole foods, is generally more acceptable and less restrictive than EEN. Earlier studies suggested that PEN alone yields lower and more variable induction efficacy; however, recent findings suggest that higher-percentage PEN (≥50% of energy) may offer improved outcomes, although results remain inconsistent and are based on relatively small and heterogeneous cohorts. This variability highlights that the “whole food” component must be carefully structured to maintain efficacy [54,55,56].

### 5.2. Crohn’s Disease Exclusion Diet (CDED)

The CDED is a structured whole-food diet that excludes UPFs, emulsifiers, and other suspect components while encouraging fruits, vegetables, and unprocessed proteins. In a pivotal randomized controlled trial, CDED combined with PEN was non-inferior to EEN for induction of remission in pediatric CD, with better tolerance and sustainability [57]. Longer-term follow-up showed higher rates of sustained remission compared with PEN alone [56]. These findings positioned CDED as an evidence-based, UPF-reduction diet with both mechanistic plausibility and clinical efficacy. Nonetheless, evidence is predominantly pediatric, and adult data remain limited.

### 5.3. Individualized Food-Based Diet (CD-TREAT)

The CD-TREAT diet reproduces the nutritional composition of EEN using whole, ordinary foods while excluding UPFs and known dietary triggers. In a pilot randomized trial involving 25 healthy adults and 5 children with active CD, CD-TREAT demonstrated anti-inflammatory and microbiota-modulating effects comparable to EEN, including significant reductions in fecal calprotectin [58]. Interpretation is limited, however, by very small sample size, short duration, and lack of diverse populations. Although still experimental, CD-TREAT highlights the feasibility of a food-based dietary therapy that minimizes UPFs while maintaining nutritional adequacy.

### 5.4. Specific Carbohydrate Diet (SCD) and Mediterranean Diet (MedDiet)

The SCD, which restricts complex carbohydrates, processed foods, and food additives, has demonstrated clinical and mucosal improvements in small pediatric cohorts with CD [59]. Similarly, a MedDiet emphasizing minimally processed plant foods, olive oil, whole grains, and fish was shown in the DINE-CD randomized trial to be non-inferior to SCD for achieving symptomatic remission, while offering superior palatability and adherence [60]. Because both diets substantially reduce UPFs by design, they illustrate the potential benefits of whole-food dietary patterns; however, long-term adherence to restrictive diets such as SCD is difficult, and evidence in adults remains inconsistent.

### 5.5. Additive-Exclusion Approaches

Targeted elimination of specific food additives has also been explored as a therapeutic strategy. A double-blind randomized trial in patients with UC in remission showed that carrageenan supplementation accelerated clinical relapse compared with a carrageenan-free diet, suggesting a potential benefit of carrageenan exclusion in susceptible individuals [32].

In CD, small pilot trials of low-microparticle diets aimed at reducing exposure to titanium dioxide (E171) and other particulate additives reported improvements in symptoms and inflammatory markers; however, these studies were conducted more than a decade ago, involve small samples, and have not been validated in modern cohorts [44]. Collectively, these studies suggest that specific UPF components may modulate inflammation, but evidence remains preliminary.

### 5.6. Guideline and Consensus Perspectives

Professional society documents increasingly acknowledge the role of diet and food processing in IBD care. The 2024 AGA Clinical Practice Update advises clinicians to counsel patients to emphasize Mediterranean-style, whole-food dietary patterns and to limit UPFs, added sugars, and salt, while noting that no single diet has consistently reduced flare rates in adults [61].

The 2025 ECCO Consensus on Dietary Management of IBD frames diet as a therapeutic modality beyond nutritional support, summarizing evidence for formula-based and food-based strategies (e.g., EEN, CDED, MedDiet) and providing practical consensus statements for clinical implementation. At the same time, it highlights evidence gaps and stops short of recommending blanket avoidance of all ultra-processed foods for every patient [62,63]. At the same time, ECCO cautions that evidence gaps remain substantial, particularly for UC, and does not endorse universal UPF avoidance for all patients.

The ESPEN Clinical Nutrition Guideline provides graded recommendations across disease phases and endorses individualized assessment, enteral nutrition for induction in CD, and whole-food dietary patterns, while warning that cultural, economic, and access-related barriers may limit feasibility in routine practice [64].

### 5.7. Therapeutic Synthesis

Therapeutic dietary approaches in IBD increasingly converge on a common principle: minimizing exposure to UPFs and certain additives. Robust evidence supports EEN and CDED in pediatric CD, with emerging, but less definitive, data for adult CD (MedDiet, SCD). Evidence for UC is limited mainly to additive-specific exclusion trials such as carrageenan restriction. Although targeting UPFs is biologically plausible, major challenges include the small size and methodological limitations of existing trials, variation in adherence, scalability of complex diets, nutritional adequacy, and long-term sustainability.

## 6. Conclusions

UPFs, characterized by refined substrates and multiple food additives, are increasingly recognized as plausible environmental contributors to IBD. Large-scale prospective studies consistently demonstrate a positive association between high UPF consumption and CD, whereas findings for UC are less consistent, highlighting disease-specific heterogeneity and the need for cautious interpretation.

Mechanistic studies predominantly in animal models implicate components typical of UPFs including emulsifiers, carrageenan, maltodextrin, fructose, microparticles, and excess dietary salt in disrupting epithelial barrier integrity, altering gut microbial composition and function, reducing beneficial metabolites, and activating innate and adaptive immune pathways. However, these effects are dose-dependent and species-specific, and their direct relevance to human IBD remains to be fully established.

Clinical data from dietary interventions further support the concept that reducing UPF exposure may offer therapeutic benefit, particularly in CD. Currently, the strongest evidence supports formula-based and structured exclusion diets such as EEN and the CDED for induction of remission in pediatric CD, while evidence for whole-food patterns like the Mediterranean diet or additive-exclusion strategies in adult IBD remains emerging, with limited sample sizes and heterogeneous outcomes requiring further validation.

## 7. Future Directions

To advance the role of diet in IBD management and prevention, future research must address several key gaps:

- Standardization of exposure: Development of harmonized definitions of UPFs and validated tools. including AI-assisted dietary monitoring and specific biomarkers of food processing, to enable cross-study comparability.
- Mechanism-focused trials: Well-designed randomized controlled trials targeting specific additives (e.g., emulsifiers, carrageenan) at physiologically relevant doses, utilizing endoscopic, histologic, and multi-omics outcomes to bridge the translational gap between animal models and human disease.
- Personalized nutrition strategies: Integration of individual microbiome characteristics, host genetics, and baseline immune signatures to tailor dietary interventions for specific IBD phenotypes.
- Long-term feasibility and safety: Rigorous evaluation of adherence, nutritional adequacy, cost-effectiveness, and real-world barriers to implementing UPF-restricted diets in diverse real-world clinical settings.
- Policy and clinical translation: Development of public health strategies for reducing population-level UPF intake, regulatory guidance for industry reformulation to minimize harmful additives, and clear pathways for integrating dietitian-led counseling into routine IBD care.

By addressing these priorities, future research may move beyond general associations to establish evidence-based, mechanism-informed, and personalized nutrition approaches that complement pharmacologic therapy and contribute to broader food policy efforts relevant to chronic inflammatory diseases.

## Figures and Tables

**Table 1:** Prospective cohort studies evaluating ultra-processed food (UPF) intake and incident inflammatory bowel disease (IBD).

Study/ Country	Sample Size/Follow-Up	Exposure Assessment	UPF Definition	Outcome	Key Findings
PURE [1] 21 countries	116,087/ median 9.7 years	Baseline FFQ	NOVA-defined UPF (servings/day)	Incident IBD	Consumption of ≥5 UPF servings/day was associated with a higher risk of incident IBD (HR 1.82; CD and UC combined). The strongest associations were observed for soft drinks, refined sweetened foods, salty snacks, and processed meats.
NHS I, NHS II, HPFS [6] USA	245,112/ 5,468,444 person-years	Repeated FFQs every 2–4 years	NOVA-defined UPF	CD, UC	Higher UPF intake was associated with an increased risk of CD in a dose–response manner. No consistent association was observed between UPF intake and UC.
NutriNet-Santé [12] France	105,832/ mean 2.3 years	Repeated web-based 24 h dietary records	Dietary UPF proportion (NOVA)	Incident IBD	No significant association was observed between UPF consumption and incident IBD. The authors noted a limited number of cases and relatively short follow-up duration.
UK Biobank [7] UK	187,854/ median 9.84 years	Web-based 24 h dietary recall questionnaires	NOVA-defined UPF intake	CD, UC; IBD-related surgery	Higher UPF consumption was associated with an increased risk of CD (HR 2.00 for highest vs. lowest intake), but not UC. Among individuals with IBD, higher UPF intake was also associated with an increased risk of IBD-related surgery.

CD, Crohn’s disease; UC, ulcerative colitis; IBD, inflammatory bowel disease; UPF, ultra-processed food; FFQ, food frequency questionnaire; HR, hazard ratio; NHS, Nurses’ Health Study; HPFS, Health Professionals Follow-up Study.

**Table 2:** Patient-based studies examining UPF intake in relation to disease activity and relapse among individuals with established IBD.

Study/ Country	Population	Design	UPF Exposure	Outcome	Key Findings
Sarbagili-Shabat 2024 [8] Israel	242 IBD patients	Cross- sectional	NOVA-defined UPF intake	Disease activity	Higher UPF intake was associated with active disease; minimally processed foods showed protective associations.
Sarbagili-Shabat 2025 [9] Israel	111 CD patients in remission	Prospective (1 year)	UPF intake tertiles (NOVA)	Clinical relapse	Higher UPF intake was associated with increased risk of relapse.
UK Biobank IBD subgroup [7] UK	Individuals with IBD	Prospective	NOVA-defined UPF intake	IBD-related surgery	Higher UPF intake was associated with increased risk of IBD-related surgery.
Christensen et al. [18] Multinational	IBD populations	Umbrella review	Various dietary exposures	Disease activity and progression	Processed food–rich diets were associated with worse outcomes, whereas whole-food–based patterns were protective.

IBD, inflammatory bowel disease; CD, Crohn’s disease; UPF, ultra-processed food.

**Table 3:** Mechanistic evidence linking UPF components and food additives to intestinal barrier dysfunction, microbiota alterations, and immune activation relevant to IBD.

Component/ Additive	Model	Dose & Exposure	Mechanistic Pathway	Key Findings	References
Emulsifiers (CMC, P80)	Mouse; human RCT	Mouse: 1.0% (*w*/*v*) in water, 12 weeks Human: CMC 15 g/day, 11 days	Mucus thinning; Microbial encroachment	Induces low-grade inflammation in WT mice; Human RCT showed microbiota depletion and reduced SCFAs.	[10,23]
Carrageenan (CGN)	Human IECs; mouse; RCT in UC	Cell: 1–10 µg/mL Human: 200 mg/day capsule	TLR4–Bcl10–NF-κB activation	Promoted epithelial inflammation in experimental models; accelerated clinical relapse in UC patients in remission.	[24,26,30,31,32]
Maltodextrin (MDX)	IECs; macrophages; IL-10^−/−^ mice	Mouse: 5% (*w*/*v*) in water	Biofilm formation; AIEC adhesion; impaired antimicrobial defense	Enhanced AIEC expansion, disrupted mucosal defense, and exacerbated colitis severity.	[33,34,35,36]
Fructose (high-fructose diets)	Mouse	High-fructose diet (e.g., 60%)	Dysbiosis; Barrier disruption	Worsened colitis; effects were reversible after dietary normalization or psyllium supplementation.	[37,38]
Titanium dioxide (E171)	Mouse; in vitro; pilot RCT	Mouse: 10 mg/kg body weight/day Human: Dietary exclusion	Microparticle uptake; Biofilm modulation; immune activation	Induced preneoplastic and inflammatory changes in animal models; pilot RCT showed reduced inflammation with a microparticle-free diet; safety concerns highlighted in regulatory assessment.	[41,43,44,45]

CMC, carboxymethylcellulose; P80, polysorbate-80; RCT, randomized controlled trial; IECs, intestinal epithelial cells; SCFAs, short-chain fatty acids; UC, ulcerative colitis; AIEC, adherent-invasive *Escherichia coli*. Note: Doses selected in the cited animal studies generally aim to mimic relevant human dietary exposures estimated from high consumption of ultra-processed foods, although translational limitations regarding species-specific metabolism and food matrix effects apply.

## Data Availability

This study is a narrative review based on previously published literature. No new data were generated, and all referenced materials are available within the cited publications.

## Abbreviations

The following abbreviations are used in this manuscript:

IBD	Inflammatory bowel disease
CD	Crohn’s disease
UC	Ulcerative colitis
UPF	Ultra-processed food
NOVA	NOVA food classification system
FFQ	Food frequency questionnaire
HR	Hazard ratio
OR	Odds ratio
RR	Relative risk
SES	Socioeconomic status
SCFA	Short-chain fatty acid
AIEC	Adherent-invasive *Escherichia coli*
CMC	Carboxymethylcellulose
P80	Polysorbate-80
CGN	Carrageenan
MDX	Maltodextrin
NNS	Non-nutritive sweetener
IEC	Intestinal epithelial cell
TLR	Toll-like receptor
NF-κB	Nuclear factor kappa B
Th17	T helper 17 cell
EFSA	European Food Safety Authority
